# Next-generation sequencing identifies altered whole blood microRNAs in neuromyelitis optica spectrum disorder which may permit discrimination from multiple sclerosis

**DOI:** 10.1186/s12974-015-0418-1

**Published:** 2015-10-31

**Authors:** Andreas Keller, Petra Leidinger, Eckart Meese, Jan Haas, Christina Backes, Ludwig Rasche, Janina R. Behrens, Catherina Pfuhl, Katharina Wakonig, René M. Gieß, Sven Jarius, Benjamin Meder, Judith Bellmann-Strobl, Friedemann Paul, Florence C. Pache, Klemens Ruprecht

**Affiliations:** Clinical Bioinformatics, Saarland University, Saarbrücken, Germany; Human Genetics, Saarland University, Homburg, Germany; Internal Medicine III, Heidelberg University Hospital, Heidelberg, Germany; Department of Neurology, Charité—Universitätsmedizin Berlin, Charitéplatz 1, 10117 Berlin, Germany; NeuroCure Clinical Research Center, Charité—Universitätsmedizin Berlin, Berlin, Germany; Clinical and Experimental Multiple Sclerosis Research Center, Charité—Universitätsmedizin Berlin, Berlin, Germany; Molecular Neuroimmunology Group, Department of Neurology, University of Heidelberg, Heidelberg, Germany; Experimental and Clinical Research Center, Max Delbrueck Center for Molecular Medicine and Charité—Universitätsmedizin Berlin, Berlin, Germany

**Keywords:** Neuromyelitis optica spectrum disorder, Multiple sclerosis, MicroRNAs, Biomarker, Whole blood, Serum, Next-generation sequencing

## Abstract

**Background:**

Neuromyelitis optica spectrum disorder (NMOSD) and multiple sclerosis (MS) have a similar clinical phenotype but represent distinct diseases, requiring different therapies. MicroRNAs (miRNAs) are short non-coding RNAs whose expression profiles can serve as diagnostic biomarkers and which may be involved in the pathophysiology of neuroinflammatory diseases. Here, we analyzed miRNA profiles in serum and whole blood of patients with NMOSD and clinically isolated syndrome (CIS)/relapsing-remitting MS (RRMS) as well as healthy controls by next-generation sequencing (NGS).

**Methods:**

MiRNA expression profiles were determined by NGS in sera of patients with aquaporin-4 antibody-positive NMOSD (*n* = 20), CIS/RRMS (*n* = 20), and healthy controls (*n* = 20) and in whole blood of patients with NMOSD (*n* = 11), CIS/RRMS (*n* = 60), and healthy controls (*n* = 43). Differentially expressed miRNAs were calculated by analysis of variance and *t* tests. All significance values were corrected for multiple testing. Selected miRNAs were validated in whole blood of patients with NMOSD (*n* = 18) and CIS/RRMS (*n* = 19) by quantitative real-time polymerase chain reaction (qRT-PCR).

**Results:**

None of 261 miRNAs detected in serum but 178 of 416 miRNAs detected in whole blood showed significantly different expression levels among the three groups. Pairwise comparisons revealed 115 (NMOSD vs. CIS/RRMS), 141 (NMOSD vs. healthy controls), and 44 (CIS/RRMS vs. healthy controls) miRNAs in whole blood with significantly different expression levels. qRT-PCR confirmed different expression levels in whole blood of patients with NMOSD and CIS/RRMS for 9 out of 10 exemplarily chosen miRNAs. In silico enrichment analysis demonstrated an accumulation of altered miRNAs in NMOSD in particular in CD15^+^ cells (i.e., neutrophils and eosinophils).

**Conclusions:**

This study identifies a set of miRNAs in whole blood, which may have the potential to discriminate NMOSD from CIS/RRMS and healthy controls. In contrast, miRNA profiles in serum do not appear to be promising diagnostic biomarkers for NMOSD. Enrichment of altered miRNAs in CD15^+^ neutrophils and eosinophils, which were previously implicated in the pathophysiology of NMOSD, suggests that miRNAs could be involved in the regulation of these cells in NMOSD.

**Electronic supplementary material:**

The online version of this article (doi:10.1186/s12974-015-0418-1) contains supplementary material, which is available to authorized users.

## Background

Neuromyelitis optica (NMO) was formerly considered a variant of multiple sclerosis (MS) [1, 2]. However, the discovery of a highly specific serum autoantibody against the aquaporin-4 (AQP4) water channel, which is present in 60–80 % of patients with NMO, clarified that NMO is a separate disease pathophysiologically distinct from MS [3–5]. While the classical NMO phenotype consists of a longitudinally extensive transverse myelitis (LETM) and optic neuritis (ON), AQP4 antibody-positive patients can also present with isolated LETM, ON, or brainstem encephalitis. This spectrum of clinical manifestations is now referred to as NMO spectrum disorder (NMOSD) [5, 6]. Because the clinical phenotype of NMOSD and MS may be similar, but each condition requires different therapeutic approaches, accurate differentiation of NMOSD and MS is highly relevant [7]. Although magnetic resonance imaging and AQP4 antibody testing aid in the differential diagnosis, misdiagnosis of NMOSD with MS is not infrequent [8, 9] and reliable biomarkers that distinguish between both entities would be desirable.

MicroRNAs (miRNAs) are single-stranded, short (20–24 nucleotide) regulatory RNAs that regulate gene expression at the posttranscriptional level. MiRNAs are present in stable form in the human blood, and blood-based miRNA expression profiles may hold promise as diagnostic biomarkers in various human diseases, including cancer and autoimmune diseases [10]. Indeed, studies performed by others and our group indicate that miRNA expression profiles determined either in whole blood or purified blood cell subtypes could play a role as potential biomarkers for MS [11–25]. Furthermore, miRNAs circulating in plasma were recently shown to be differentially expressed in different stages of MS (relapsing-remitting MS (RRMS) vs. secondary progressive MS) and were thus proposed as an easily accessible blood-based biomarker to monitor MS [26].

MiRNA profiles have hitherto not been investigated in NMOSD and their role in the differentiation of NMOSD, and MS has not been explored so far. A possible role of miRNAs in the pathophysiology of NMOSD likewise remains elusive. We here report the results of a systematic comparative analysis of miRNA profiles in serum and whole blood of patients with NMOSD and clinically isolated syndrome (CIS)/RRMS as well as healthy controls using next-generation sequencing.

## Methods

### Ethics, consent, and permissions

The study was performed in accordance with the Declaration of Helsinki and was approved by the institutional review board, Charité—Universitätsmedizin Berlin, (EA1/131/09). All participants provided written informed consent.

### Serum and whole blood samples of patients and healthy controls

Between May and November 2013, serum (collected in Sarstedt serum tubes [REF 02.1063]; Sarstedt, Nürmbrecht, Germany) and whole blood samples (collected in PAXgene Blood RNA tubes; Becton Dickinson, Heidelberg, Germany) were obtained by peripheral venipuncture of a total of 20 patients with NMOSD (age >18 years), presenting with LETM and ON (*n* = 11), LETM and brainstem encephalitis (*n* = 3), isolated LETM (*n* = 4), or isolated ON (*n* = 2). All patients with NMOSD had serum antibodies to AQP4 as determined by a cell-based assay [27]. Serum and whole blood samples were likewise obtained from a total of 44 participants of an ongoing prospective observational study of patients with early MS (Berlin CIS cohort, NCT01371071), which started recruitment in January 2011. Inclusion criteria were age >18 years, a first clinical event suggestive of central nervous system demyelination (CIS) not meeting the McDonald 2010 criteria for RRMS [28] within 6 months before study inclusion, or a diagnosis of RRMS according to the McDonald 2010 criteria within 24 months before study inclusion. To increase the number of whole blood miRNA profiles of patients with CIS/RRMS, we additionally used miRNA profiles of 16 further patients with CIS/RRMS obtained in a previous project, in which exactly the same methodology as in the present work was applied [25]. In addition, serum samples were also collected from 20 healthy controls. Furthermore, we included whole blood miRNA profiles of a total of 43 healthy controls obtained in two previous projects (*n* = 21 from Keller et al. [25] and *n* = 22 from Leidinger et al. [29]), in which, again, exactly the same methodology as in the present work was applied. Except for the 22 healthy control samples from Leidinger et al. [29], which were purchased from PrecisionMed (San Diego, CA, USA), all patients with NMOSD and CIS/RRMS and healthy controls were recruited at the Department of Neurology and NeuroCure Clinical Research Center, Charité—Universitätsmedizin Berlin. Serum and whole blood samples were withdrawn in parallel and processed at the NeuroCure Clinical Research Center according to standard operating procedures and stored at −80 °C. Coded serum samples were shipped on dry ice to the Department of Internal Medicine III, Heidelberg University, and coded whole blood samples were shipped on dry ice to the Department of Human Genetics, Saarland University, for further blinded processing.

### RNA isolation from serum

The *mir*VANA PARIS Kit Ambion (AM1556) was used to isolate RNA from 200 μl of serum. In addition to the manufacturer’s protocol, precipitation was also performed using 2 M ammonium acetate. To gain sufficient amounts of miRNA, the isolation procedure was performed twice and eluates were pooled.

### Library preparation and next-generation sequencing of serum samples

For the library preparation, 6 μl of the eluates from the serum RNA isolation was used. Preparation was performed following the protocol of the TruSeq Small RNA Sample Prep Kit (Illumina). To reduce adapter dimerization, we only used half the amount of adapters during preparation. Concentration of the ready prepped libraries was measured on the Bioanalyzer using the High Sensitivity Chip. Libraries were then pooled in batches of six samples in equal amounts and clustered with a concentration of 18 pmol in one lane each of a single read flowcell using the cBot (Illumina). Sequencing of 50 cycles was performed on a HiSeq 2000 (Illumina). Demultiplexing of the raw sequencing data and generation of the fastq files were done using CASAVA v.1.8.2.

### RNA isolation from whole blood and next-generation sequencing of whole blood samples

Total RNA including miRNA was isolated from whole blood samples using the PAXgene Blood miRNA Kit (Qiagen, Hilden, Germany), and next-generation sequencing was performed on a HiSeq2000 System (Illumina) exactly as previously described in detail [25].

### Quantitative real-time polymerase chain reaction

Quantitative real-time polymerase chain reaction (qRT-PCR) on whole blood was performed using the miScript PCR System according to the manufacturer’s recommendations (Qiagen). Samples included in the qRT-PCR study were analyzed as individual and not as pooled samples. For whole blood samples, the small RNA RNU48 was used as endogenous control for normalization according to the ∆Ct method [30]. The mean ± standard deviation Ct value of RNU48 of the 37 samples analyzed was 19.89 ± 0.78.

### Bioinformatics analysis

For matching raw NGS reads to miRNAs, the miRDeep algorithm was applied [31]. Subsequent calculations were carried out using the freely available statistical programming environment R (version 3.0.2). To make NGS raw read counts more comparable, standard quantile normalization was used as implemented in the *normalize.quantiles* function. Analysis of variance (ANOVA) was carried out by the *anova* function. Pairwise *t* tests were carried out using the *t.test* function. Adjustment for multiple testing was performed by controlling the false discovery rate according to the approach of Benjamini and Hochberg. Besides *t* tests, the area under the receiver operator characteristic curve (AUC value) was computed to determine how well the miRNAs separate between two groups. Box and whisker plots were generated by using the *boxplot* function. For calculating volcano plots, the log2 of the mean expression was calculated and plotted against the log10 *p* value (raw *p* values were used for this purpose). Different expression levels in the qRT-PCR analysis were assessed by *t* test and Mann-Whitney test. Gender distribution between the groups of patients with NMOSD, CIS/RRMS, and healthy controls was assessed by a 3 × 2 Fisher’s exact test. Age differences between groups were assessed by Kruskal-Wallis or Mann-Whitney test. Moreover, the miRNACon tool (freely available at http://www.ccb.uni-saarland.de/mirnacon/) was applied in order to identify miRNAs that are potentially influenced by age or gender [32]. Finally, we used our tool miEAA (http://www.ccb.uni-saarland.de/mieaa_tool/) to further characterize the association of sets of miRNAs with pathways, functional categories, diseases, tissues, or cell types. MiEAA is based on GeneTrail [33] and performs standard enrichment analyses like over-representation analysis or gene set enrichment analysis in the context of miRNAs. This way, we identify significantly enriched miRNA categories where we find more miRNAs in our input set than expected by chance. If not mentioned otherwise, adjusted *p* values <0.05 were considered significant.

## Results

### Participants

Demographic and clinical characteristics of patients with NMOSD, CIS/RRMS, and healthy controls studied in the different parts of this work are summarized in Table 1. All NMOSD patients were in remission at the time of sampling, and no one had experienced a relapse within the prior 4 months. There were no significant differences in the gender distributions between the various groups in the different analyses. However, patients with NMOSD were significantly older than patients with CIS/RRMS, which is in accordance with the higher median age at onset in AQP4-IgG-positive NMOSD compared to MS [5] and healthy controls in the serum NGS study. Furthermore, healthy controls were significantly older than patients with NMOSD and CIS/RRMS in the whole blood NGS study. While the vast majority of patients with CIS/RRMS were untreated, most patients with NMOSD were treated with immunotherapy, reflecting the more severe course of NMOSD compared to MS [8].

**Table 1:** Demographic and clinical characteristics of the patients with NMOSD and CIS/RRMS as well as of healthy controls included in this study

Serum (NGS)				
	NMOSD (*n* = 20)	CIS/RRMS (*n* = 20; 16/4)	Healthy controls (*n* = 20)	*p* value^a^
Females/males (% female)	18/2 (90)	13/7 (65)	13/7 (65)	0.13
Median age, y (range)	49.5 (18–75)	33 (20–41)	32 (21–42)	0.004
Median EDSS (range)	4 (1–9)	1.25 (0–2.5)	–	–
Immunotherapy (number)	AZA (7) AZA + S (2) MTX + Quensyl (1) GLAT (1) RTX (6) MMF (1) None (2)	Interferon-beta (1) none (19)	–	–
Whole blood (NGS)				
	NMOSD (*n* = 11)	CIS/RRMS (*n* = 60; 50/10)^b^	Healthy controls (*n* = 43)^c^	*p* value
Females/males (% female)	10/1 (91)	38/22 (63)	23/20 (53.5)	0.07
Median age, y (range)	34 (21–75)	31.5 (19–50)	60 (21–83)	0.0003
Median EDSS (range)	4 (1–9)	1.5 (0–3.5)^d^	–	–
Immunotherapy	AZA (3) AZA + S (1) MTX + Quensyl (1) GLAT (1) RTX (4) None (1)	Interferon-beta (1) None (59)	–	–
qRT-PCR validation study				
	NMOSD (*n* = 19)	CIS/RRMS (*n* = 19; 15/4)	–	*p* value
Females/males (% female)	17/2 (85)	17/2 (85)	–	1.0
Median age, y (range)	50 (21–75)	41 (23–51)	–	0.1
Median EDSS (range)	3.75 (1–9)	2 (0–3.5)	–	–
Immunotherapy (number)	AZA (7) AZA + S (2) MTX + Quensyl (1) GLAT (1) RTX (5) MMF (1) None (2)	GLAT (2) Interferon-beta (4) None (13)	–	–

*NGS* next-generation sequencing, *y* years, *EDSS* expanded disability status scale, *AZA* azathioprine, *AZA + S* azathioprine and corticosteroids, *MTX* methotrexate, *GLAT* glatiramer acetate, *RTX* rituximab, *MMF* mycophenolate mofetil, *CIS* clinically isolated syndrome, *RRMS* relapsing-remitting multiple sclerosis, *NMOSD* neuromyelitis optica spectrum disorder

^a^Gender distribution was assessed by 2 × 3 or 2 × 2 Fisher exact test and age differences by Kruskal-Wallis or Mann-Whitney tests

^b^Forty-four of the 60 patients with CIS/RRMS included in this work were recruited in the present study and 16 miRNA expression profiles were from a previous work [25]

^c^Blood miRNA profiles from the 43 healthy controls were obtained in two previous projects (*n* = 21 from Keller et al. [25] and *n* = 22 from Leidinger et al. [29])

^d^EDSS values were only available for the 44 patients recruited in the present study

### MiRNA profiles in serum

**Fig. 1:**
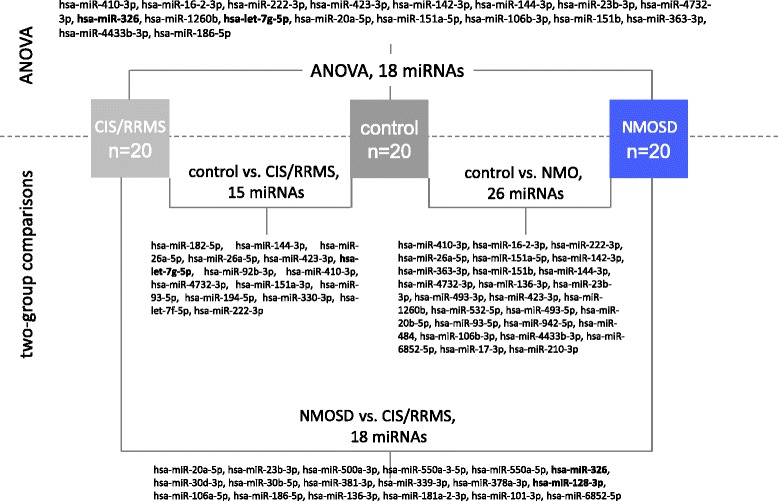
Synopsis of results of the serum NGS study. MiRNA expression profiles were compared in sera of 20 patients with NMOSD, 20 patients with CIS/RRMS, and 20 healthy controls. The *upper part* of the figure lists the 18 miRNAs with significant unadjusted *p* values as detected by a three-group comparison by ANOVA. The *lower part* lists the results of two-group comparisons between the different groups. *CIS* clinically isolated syndrome, *RRMS* relapsing-remitting multiple sclerosis, *NMOSD* neuromyelitis optica spectrum disorder, *ANOVA* analysis of variance

**Fig. 2:**
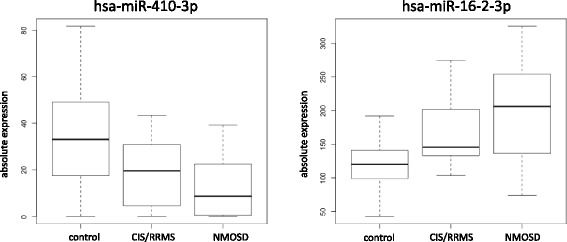
Expression levels of the two most significantly different serum miRNAs. The absolute expression levels of the two most significantly deregulated serum miRNAs, hsa-miR-410-3p and hsa-miR-16-2-3p, in the three-group comparison of patients with NMOSD, CIS/RRMS, and healthy controls by ANOVA are shown as *boxplots. CIS* clinically isolated syndrome, *RRMS* relapsing-remitting multiple sclerosis, *NMOSD* neuromyelitis optica spectrum disorder

Intrigued by recent findings suggesting that non-cell-associated miRNAs in plasma may be capable of differentiating between different stages of MS [26], we investigated whether miRNA profiles in serum may have a similar diagnostic value. We thus analyzed miRNA profiles in sera of patients with NMOSD (*n* = 20), CIS/RRMS (*n* = 20), and healthy controls (*n* = 20) by genome-wide sequencing of all miRNAs on the Illumina HiSeq2000 platform. The raw data were pre-processed as described in the “Methods” section and normalized using quantile normalization. Additionally, we excluded very lowly (less than 100 reads) and very highly abundant miRNAs (>50,000 reads). Taken all three groups together, this identified 286 miRNAs stably expressed in serum. These miRNAs are listed with the respective mean value, median value, and standard deviation in the three groups in Additional file 1. After removing duplicated mature miRNAs from different precursors, 261 expressed miRNAs remained. We next carried out ANOVA to identify miRNAs, which are differently expressed in one of the three groups. As presented in Fig. 1, this revealed 18 miRNAs with significantly different expression levels in one of the three groups (raw *p* value <0.05). However, given the large number of miRNAs analyzed, none of the reported miRNAs remained significant after correction for multiple testing. Nevertheless, several miRNAs showed interesting patterns. The two most significant miRNAs, hsa-miR-410-3p (raw *p* = 0.0005) and hsa-miR-16-2-3p (raw *p* = 0.004), are shown as box plots in Fig. 2. While hsa-miR-410-3p was lower in NMOSD (average of 12 counts) than in CIS/RRMS (average of 19 counts) and controls (average of 36 counts), hsa-miR-16-2-3p was higher in NMOSD (average of 207 counts) than in CIS/RRMS (average of 162 counts) and controls (average of 124 counts).

To identify differentially expressed miRNAs, which discriminate between two groups, we next carried out pairwise comparisons using *t* tests. When comparing healthy controls vs. CIS/RRMS and healthy controls vs. NMOSD, we found 15 and 26 significantly different miRNAs (raw *p* values of two-sided *t* tests <0.05), respectively. Comparison of CIS/RRMS vs. NMOSD detected 18 significantly different miRNAs. The respective miRNAs are listed in Fig. 1 (lower part). However, again, none of the miRNAs remained significant after correction for multiple testing. Detailed information on the pairwise comparisons including raw and adjusted *p* values as well as AUC values for all discovered serum miRNAs including the duplicates from different precursors are provided in Additional file 2.

### MiRNA profiles in whole blood

**Fig. 3:**
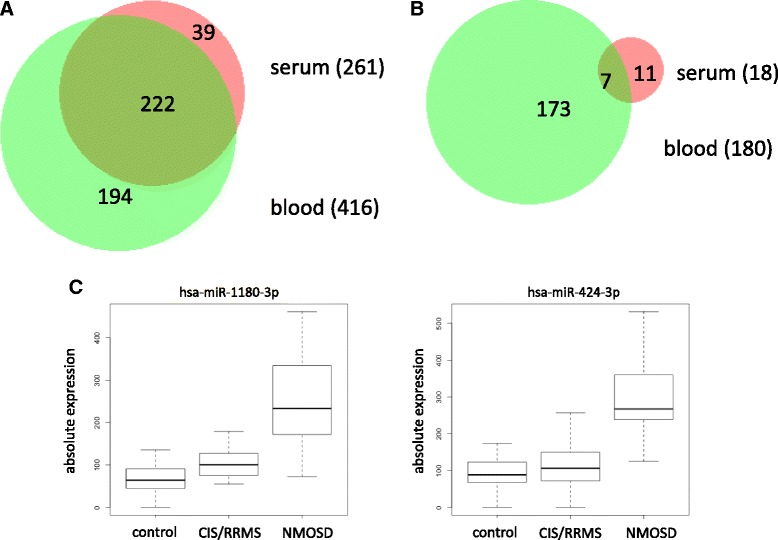
Overview of results from miRNA expression profiling in whole blood. **a** Venn diagram showing the overlap of all miRNAs detected in serum and whole blood after removal of duplicated mature miRNAs. **b** Venn diagram showing the overlap of miRNAs found to be deregulated in serum and whole blood (raw *p* values <0.05) in the three-group comparison of patients with NMOSD, CIS/RRMS, and healthy controls by ANOVA. **c** The absolute expression levels of the two most significantly deregulated whole blood miRNAs, has-miR-1180-3p and has-miR-424-3p, in the three-group comparison (ANOVA) of patients with NMOSD, CIS/RRMS, and healthy controls are shown as *boxplots. CIS* clinically isolated syndrome, *RRMS* relapsing-remitting multiple sclerosis, *NMOSD* neuromyelitis optica spectrum disorder, *ANOVA* analysis of variance

Compared to the results for serum outlined above, we previously had observed more significant results in our research on miRNA profiles in whole blood of patients with CIS/RRMS and healthy controls [10, 12, 25]. Thus, we went on to carry out miRNA sequencing in whole blood samples. In this analysis, we included 11 patients with NMOSD, 60 patients with CIS/RRMS, and 43 healthy controls. Raw miRNA expression data were processed as described in the “Methods” section. In the whole blood samples, we detected, in total, 477 miRNAs corresponding to 416 unique mature miRNAs after excluding duplicated mature miRNAs from different precursors. Of these, 222 were also detected in serum as shown in the Venn diagram in Fig. 3a. In analogy to the approach pursued for serum miRNAs, we next searched for miRNAs in whole blood that differ in one of the three groups using ANOVA. This computation identified 211 miRNAs (corresponding to 180 unique mature miRNAs after removing duplicates from different precursors), which were significantly altered in one of the three groups prior to adjustment for multiple testing. Of these, 178 remained significant (*p* < 0.05) after adjustment for multiple testing. The 477 blood-borne miRNAs are listed with the respective mean value, median value, and standard deviation in the three groups in Additional file 3.

Since for the serum analysis no significant miRNAs remained after adjustment for multiple testing, we compared the significant miRNAs for blood and serum prior to adjustment. Remarkably, of the 18 miRNAs found to be different (raw *p* < 0.05) among the three groups by ANOVA in serum, seven miRNAs were likewise discovered in the ANOVA of blood samples, as detailed in Fig. 3b. The two most significant miRNAs in the whole blood ANOVA, hsa-miR-1180-3p (adjusted *p* value 10^−12^) and hsa-miR-424-3p (adjusted *p* value 10^−10^), are shown as boxplots in Fig. 3c. Both of these miRNAs were higher in patients with NMOSD than in patients with CIS/RRMS and healthy controls.

Next, we calculated significant values for the pairwise comparison of whole blood miRNAs in patients with NMOSD and CIS/RRMS. This identified 176 significant miRNAs prior to adjustment and still as much as 115 significant (*p* < 0.05) miRNAs following adjustment for multiple testing. The most significantly different miRNA was hsa-miR-30b-5p, which showed a 2.8-fold upregulation in CIS/RRMS vs. NMOSD patients with corresponding raw and adjusted *p* values of 3 × 10^−9^ and 8 × 10^−7^ and an AUC value of 0.9 (see Additional file 4 for details).

In the comparison of NMOSD vs. healthy controls, 190 miRNAs were discovered as differentially expressed prior to adjustment for multiple testing. Following adjustment, still 141 miRNAs remained significant. Interestingly, 92 of those miRNAs were significantly higher in healthy controls. Among the top 25 miRNAs, just a single miRNA, hsa-miR-425-5p, was higher in NMOSD as compared to healthy controls. The most dysregulated miRNA was hsa-miR-15b-3p. This miRNA was more than threefold more abundant in controls resulting in raw and adjusted *p* values of 6 × 10^−12^ and 3 × 10^−9^, respectively, and an AUC value of 0.98 (see Additional file 4 for details). Of note, hsa-miR-15b-3p was also the second most significant miRNA in the comparison of NMOSD vs. CIS/RRMS.

Finally, a comparison of patients with CIS/RRMS and healthy controls revealed 112 miRNAs that were significantly dysregulated between the two groups. Following adjustment for multiple testing, 44 miRNAs remained significant. Here, the most dysregulated miRNA was hsa-miR-425-3p. This miRNA was threefold higher in CIS/RRMS patients, resulting in raw and adjusted *p* values of 3 × 10^−14^ and 2 × 10^−12^, respectively. The corresponding AUC value was 0.89. All pairwise comparisons are provided with all raw as well as adjusted *p* values, fold changes, and AUC values in Additional file 4.

**Fig. 4:**
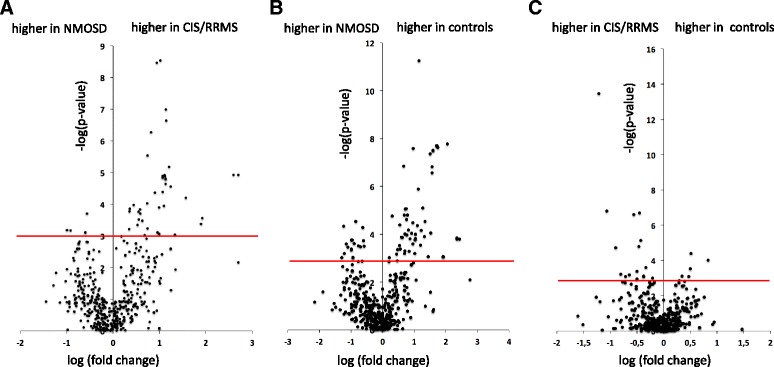
Volcano plots demonstrating different miRNA expression levels in whole blood in two-group comparisons. *Volcano plots* showing the log2 fold differences of the average miRNA expression in whole blood on the *x*-axis and the negative decade logarithm of the unadjusted *p* value on the *y*-axis. **a** Patients with NMOSD vs. patients with CIS/RRMS, **b** patients with NMOSD vs. healthy controls, **c** patients with CIS/RRMS vs. healthy controls. The *horizontal lines* indicate a *p* value of 10^−3^. *CIS* clinically isolated syndrome, *RRMS* relapsing-remitting multiple sclerosis, *NMOSD* neuromyelitis optica spectrum disorder

For a graphical representation of the extent and direction of whole blood miRNA dysregulation between the different groups, we calculated volcano plots for all three pairwise comparisons, showing the log2 fold differences of the average miRNA expression between groups on the *x*-axis and the negative decade logarithm of the *p* value on the *y*-axis. Overall, this demonstrated that more miRNAs were higher expressed in patients with CIS/RRMS than in patients with NMOSD (Fig. 4a). Indeed, setting a raw *p* value threshold of 10^−3^, 45 miRNAs were higher in CIS/RRMS than in NMOSD (upper right part of Fig. 4a), while only five miRNAs were higher in NMOSD than in CIS/RRMS (upper left part of Fig. 4a). Similarly, at a *p* value threshold of 10^−3^, more miRNAs had higher expression levels in healthy controls than in NMOSD patients (59 vs. 20; Fig. 4b). In contrast, in the comparison of CIS/RRMS and healthy controls, 15 miRNAs showed higher levels in CIS/RRMS patients but only 5 miRNAs were higher in controls (Fig. 4c).

### qRT-PCR validation

**Fig. 5:**
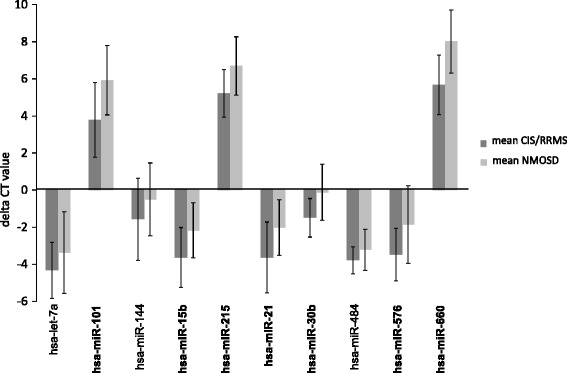
qRT-PCR validation study of differentially expressed whole blood miRNAs in patients with NMOSD and CIS/RRMS. The figure shows the absolute delta CT (target miRNA—housekeeping RNA (RNU48)) values for 10 miRNAs in the groups of patients with NMOSD (*n* = 18) and CIS/RRMS (*n* = 19). Note that higher delta CT values indicate lower expression levels. *Bars* represent the mean ± standard deviation. Expression levels of miRNAs indicated in bold were significantly different (*p* < 0.05). *CIS* clinically isolated syndrome, *RRMS* relapsing-remitting multiple sclerosis, *NMOSD* neuromyelitis optica spectrum disorder

We next aimed to validate the findings obtained in the whole blood NGS study by qRT-PCR. As the main goal of this work was to identify miRNAs that discriminate between patients with NMOSD and CIS/RRMS, we validated the whole blood NGS findings for these two patient groups. We included 19 patients with NMOSD and 19 patients with CIS/RRMS in the qRT-PCR study. One NMOSD sample had to be excluded because the amount of RNA was insufficient. Thus, a total of 18 patients with NMOSD and 19 patients with CIS/RRMS samples could be analyzed by qRT-PCR. We chose 10 miRNAs for the qRT-PCR validation study. Besides the three most significantly different miRNAs in the two-group comparison between patients with NMOSD and CIS/RRMS, hsa-miR-30b-5p, hsa-miR-15b-3p, and hsa-miR-576-5p, we selected further miRNAs with a range of different expression levels: hsa-miR-660-5p, hsa-let-7a-5p, hsa-miR-101-3p, hsa-miR-144-5p, hsa-miR-21-5p, and hsa-miR-215-5p. While all of these nine miRNAs were lower in patients with NMOSD than in patients with CIS/RRMS in the whole blood NGS study, we also included one miRNA (hsa-miR-484) with a moderately higher expression in NMOSD as compared to CIS/RRMS. Results of the qRT-PCR validation are shown in Fig. 5. The qRT-PCR study clearly confirmed the lower expression of hsa-miR-30b-5p, hsa-miR-15b-3p, hsa-miR-576-5p, hsa-miR-660-5p, hsa-let-7a-5p, hsa-miR-101-3p, hsa-miR-144-5p, hsa-miR-21-5p, and hsa-miR-215-5p in whole blood in patients with NMOSD as compared to patients with CIS/RRMS. In contrast to the whole blood NGS study, hsa-miR-484 levels were slightly, albeit not significantly, lower in patients with NMOSD compared to patients with CIS/RRMS in the qRT-PCR study. Except for let-7a-5p, hsa-miR-144-5p, and hsa-miR-484, all differences were significant (*t* test). Since the data were not normally distributed in all cases, we also calculated *p* values with the non-parametric Mann-Whitney test, which revealed the same results.

Because the miRNAs validated in the qRT-PCR study may represent potential candidates for clinical applications, we used the miRNACon tool, which we developed with the aim to discover potentially confounding variables on miRNA expression levels, to study potential confounding influences of age and gender on the expression levels of these miRNAs. This analysis demonstrated that the nine miRNAs in the signature are not dependent on gender, and just one single miRNAs showed an age dependence, namely hsa-miR-101-3p. The results of the miRNACon analysis are presented in Additional file 5.

### Enrichment analysis of miRNAs

To discover potential enrichment of miRNAs on pathways, in functional categories, or with respect to diseases, tissues, or cell types, we employed our miRNA set enrichment tool miEAA, which is freely available at http://www.ccb.uni-saarland.de/mieaa_tool/. Altogether, we thereby explored over 15,000 functional miRNA sets and analyzed whether the 141 miRNAs from the comparison NMOSD vs. healthy controls and the 115 significant miRNAs from the comparison NMOSD vs. CIS/RRMS accumulate in certain categories. Categories were considered to be enriched if they contained at least two miRNAs and had an adjusted *p* value below 0.05. The miRNA sets included in this analysis are partially direct miRNA sets but likewise contain sources from databases that also include miRNA target genes, such as miRWalk.

For the 141 miRNAs differentially expressed in NMOSD patients as compared to controls, we found the most substantial enrichment in CD15^+^ cells (adjusted *p* value 4.9 × 10^−16^; expected 25 miRNAs by chance, found 62 miRNAs). Furthermore, these miRNAs were likewise significantly enriched in CD14^+^, CD56^+^, CD3^+^, and CD19^+^ cells. Regarding disease associations, we found many unspecific hits including several cancer entities but also autoimmune diseases (expected 11, discovered 22, *p* = 0.005) and neurodegenerative diseases (data source KEGG: expected 30, discovered 44, *p* = 0.0006; data source miRWalk: expected 17, discovered 28, *p* = 0.005). Regarding biochemical pathways, we found very strong enrichment of several paths from KEGG and Biocarta, many of them connected to the immune system. The most significant pathways were calcium signaling (KEGG) and NO2IL12 (Biocarta). Other examples include the T helper and B cell pathway (Biocarta) or the IFNG pathway (Biocarta). Additionally, we discovered six target genes that were significantly enriched: IRAK1, FBXW2, SMARCD1, HMMR, SCMH1, and BAZ1B. All results of this in silico enrichment analysis are provided in Additional file 6.

For the 115 miRNAs from the comparison NMOSD vs. CIS/RRMS patients, we analogously carried out the enrichment analysis. Regarding cell types, again, CD15^+^ cells showed highest enrichment (adjusted *p* value 8.5 × 10^−11^; expected 19 miRNAs by chance, found 46 miRNAs), followed by CD14^+^, CD56^+^, CD19^+^, and CD3^+^ cells. With respect to diseases, we again found many unspecific hits but still, autoimmune diseases (expected 9, discovered 19, *p* = 0.006) and neurodegenerative diseases (data source KEGG: expected 24, discovered 35, *p* = 0.003; data source miRWalk: expected 13, discovered 25, *p* = 0.003) were significant. With respect to pathways, calcium signaling was still significant, but in this analysis, it just ranked on position 42. The most significant paths were the LDL and the ETS pathways from Biocarta. The T helper, B cell, IL17, and IL6 pathways were among the significant paths (Biocarta) as well. We also found a total of nine target genes that were significantly enriched: TNRC6B, RPL4, BAZ1B, IRS4, APP, SPATA2, NCKAP5L, TRIM33, and AGO2. Only BAZ1B has already been discovered in the first enrichment analysis. All results of this analysis are detailed in Additional file 7.

For the serum samples we had not discovered significant miRNAs in the ANOVA following adjustment for multiple testing. Nevertheless, we assessed whether we could discover potential enrichment of the most significant miRNAs prior to adjustment in an exploratory fashion. In order to minimize a potential bias due to different miRNA set size, we selected the 178 most significant miRNAs, matching the number of blood-borne miRNAs. Here, our analysis did not yield any significant results, providing further evidence for the higher relevance of miRNAs in whole blood.

## Discussion

Here, we report the results of a systematic comparative study of both miRNA profiles in serum and whole blood of patients with NMOSD, CIS/RRMS, and healthy controls. The key findings of this work are (i) an absence of any significantly different serum miRNAs after adjustment for multiple testing, (ii) the detection of a number of highly significantly deregulated miRNAs in whole blood in the three-group and the two-group comparisons of patients with NMOSD, CIS/RRMS, and healthy controls, (iii) the validation of selected whole blood miRNAs deregulated between patients with NMOSD and CIS/RRMS by qRT-PCR, and (iv) the identification of cell types, in particular, CD15^+^ neutrophils and eosinophils, and pathways in which deregulated whole blood miRNAs are enriched.

While miRNA expression profiles were previously often investigated in whole blood or leukocyte subsets, recent studies suggested that also non-cell-associated miRNAs circulating in blood and measured in serum or plasma could serve as biomarkers for cancer, inflammation, and neurological diseases, including MS [26, 34–37]. Therefore, we initially set out to analyze miRNA expression profiles in serum of patients with NMOSD, CIS/RRMS, and healthy controls. Because the pathogenesis of AQP4 antibody-negative NMOSD spectrum disorders (NMOSD) may be different from that of AQP4 antibody-positive NMOSD [8, 38], we aimed to reduce inhomogeneity within the group of patients with NMOSD and purposefully included only AQP4 antibody-positive patients in our study. The 261 miRNAs detected across all three groups in the serum NGS study confirm that miRNAs are present over a broad range of expression levels in human serum and define the landscape of human serum miRNAs. Likewise, the detectability of miRNAs in serum suggests that these miRNAs could in principle serve as disease biomarkers. Nevertheless, since none of the observed differences of the expression levels of serum miRNAs in the three-group and the two-group comparisons remained significant after correction for multiple testing, the potential of serum miRNA profiles as biomarkers for differentiation of NMOSD, CIS/RRMS, and healthy controls appears questionable.

Because more miRNAs were detectable in whole blood (*n* = 416) than in serum (*n* = 261), not all miRNAs expressed in blood cells appear to be released into serum. Alternatively, the concentration of some miRNAs in serum could also be below the detection limit of NGS. Interestingly, few miRNAs were only detected in serum but not in whole blood, compatible with an enhanced secretion of these miRNAs into serum.

MiRNA profiling by NGS in whole blood samples identified a set of differentially expressed miRNAs in the three-group comparison and in the two-group comparisons, which remained significant after correction for multiple testing. This suggests that whole blood is a more appropriate biospecimen for analyses of miRNAs as biomarkers for neuroinflammatory diseases than serum. Indeed, the high AUC values of the miRNAs, which separated best between patients with NMOSD and CIS/RRMS (hsa-miR-30b-5p; AUC 0.9) and patients with NMOSD and healthy controls (hsa-miR-15b-3p; AUC 0.98), indicate that the expression levels of these miRNAs in whole blood may potentially discriminate patients with NMOSD from patients with CIS/RRMS and healthy controls. Interestingly, hsa-miR-15b-3p was not only the most significantly different miRNA when comparing NMOSD and healthy controls but was also the miRNA that discriminated second best between NMOSD and CIS/RRMS. Downregulated expression of miR15b-3p in whole blood may thus be specifically associated with NMOSD. In both comparisons, NMOSD vs. healthy controls and NMOSD vs. CIS/RRMS, clearly more miRNAs were lower in NMOSD than in CIS/RRMS or healthy controls (Fig. 4a, b) among the significantly deregulated miRNAs. Although the reasons for the downregulation of a number of miRNAs in NMOSD are unclear, this again differentiates NMOSD from CIS/RRMS, where most of the significantly deregulated miRNAs were higher in patients than healthy controls. Altogether, the different miRNA profiles in patients with NMOSD and CIS/RRMS further support the notion that NMOSD and CIS/RRMS represent different diseases.

The different miRNA expression levels in patients with NMOSD and CIS/RRMS obtained in the whole blood NGS study could be confirmed by qRT-PCR validation for 9 out of 10 exemplarily chosen miRNAs, 7 of which (including hsa-miR-15b-3p) reached statistical significance. Findings from NGS were thus reproducible by a different method, underscoring the validity of the NGS results. Nevertheless, the absolute fold differences of the top ranking miRNAs between the different groups in the NGS and qRT-PCR studies were only about threefold, and the standard deviations between groups overlapped. Further studies in independent cohorts will therefore be mandatory to corroborate the value of the miRNAs identified in this work as diagnostic biomarkers for NMOSD.

In addition to their potential role as biomarkers, the miRNAs found to be deregulated in NMOSD could be of relevance in the pathophysiology of NMOSD. An in silico enrichment analysis demonstrated, for both comparisons NMOSD vs. CIS/RRMS and NMOSD vs. controls, a highly significant enrichment of the miRNAs deregulated in NMOSD in immune cells (CD15^+^, CD14^+^, CD56^+^, CD19^+^, and CD3^+^ cells) of relevance for NMOSD. While enrichment in these cells was not unexpected as we studied whole blood containing immune cells, quite remarkably, for both comparisons, the most significant enrichment was observed for CD15^+^ cells, i.e., neutrophils and eosinophils [39, 40]. In contrast to MS, neutrophils and eosinophils are frequently found in the cerebrospinal fluid of patients with NMOSD and are known to play an important role in the pathogenesis of NMOSD lesions [41–43]. Our findings are thus consistent with an altered function of neutrophils and eosinophils in NMOSD and suggest that miRNAs may be involved in the pathophysiology of neutrophils/eosinophils in NMOSD. However, as the functional effects of the miRNAs with altered expression profiles in NMOSD are just partially understood, the precise molecular mechanisms underlying a potential role of these miRNAs in NMOSD and in the regulation of neutrophils/eosinophils remain to be explored.

Limitations of this work are the limited number of patients with NMOSD in the whole blood NGS study and a possible confounding role of immunotherapy in NMOSD patients. However, the results obtained in the whole blood NGS study could clearly be confirmed in a larger number of patients with NMOSD in the qRT-PCR validation study, suggesting that findings obtained in a smaller number of patients are reproducible in larger cohorts. Furthermore, clinical characteristics of the patients included in this study could influence miRNA expression levels, too. While this study was not specifically designed to investigate the association of clinical characteristics with miRNA expression levels, exploratory analyses did not show an association of disease duration, the number of previous relapses, and the time since last relapse with whole blood miRNA expression levels in patients with CIS/RRMS (clinical data available for *n* = 44 patients) or NMOSD (*n* = 11).

Among the strengths of this study is the application of NGS technology, which in contrast to more traditional methods of miRNA profiling (miRNA microarrays and qRT-PCR), that are necessarily restricted to a limited number of miRNA targets, has the advantage of allowing for the unbiased detection of all known and even novel miRNAs. Thus, NGS is currently one of the most comprehensive methodological approaches to determine miRNA expression profiles [44, 45]. Another strength is that *p* values were rigorously adjusted for multiple testing, which reduces the risk of false positive findings inherent to the high number of statistical comparisons typically performed in miRNA profiling studies [44].

## Conclusions

In conclusion, this study identifies a set of differentially expressed miRNAs in whole blood, which may discriminate patients with NMOSD from patients with CIS/RRMS and healthy controls. In contrast, miRNAs expressed in serum do not appear to be promising diagnostic biomarkers for NMOSD. Although the confirmation of the NGS results for 9 out of 10 exemplarily chosen miRNAs by qRT-PCR argues for the robustness of the findings, it will be important to reproduce our results in independent patient cohorts. Enrichment of miRNAs differentially expressed in NMOSD in CD15^+^ neutrophils/eosinophils suggests that miRNAs could be involved in the regulation of neutrophils/eosinophils in NMOSD, immune cell subsets that have previously indeed been implicated in the immunopathogenesis of that condition [41–43]. Future studies should clarify the molecular mechanisms through which the miRNAs identified in this work may contribute to the pathophysiology of NMOSD.

## Additional files

Additional file 1:
**ANOVA of miRNAs in serum.**
Additional file 2:
**Pairwise group comparisons of miRNAs in serum.**
Additional file 3:
**ANOVA of miRNAs in whole blood.**
Additional file 4:
**Pairwise group comparisons of miRNAs in whole blood.**
Additional file 5:
**Results of miRNACon analysis.**
Additional file 6:
**miEAA analysis of miRNAs differentially expressed in NMOSD and healthy controls.**
Additional file 7:
**miEAA analysis of miRNAs differentially expressed in NMOSD and CIS/RRMS.**ᅟ
