# Supplementary figures and images for: Next-generation sequencing identifies altered whole blood microRNAs in neuromyelitis optica spectrum disorder which may permit discrimination from multiple sclerosis
Source: J Neuroinflammation. 2015 Oct 31;12:196. doi: 10.1186/s12974-015-0418-1 (PMC4628234; doi:10.1186/s12974-015-0418-1)

## Slide 1
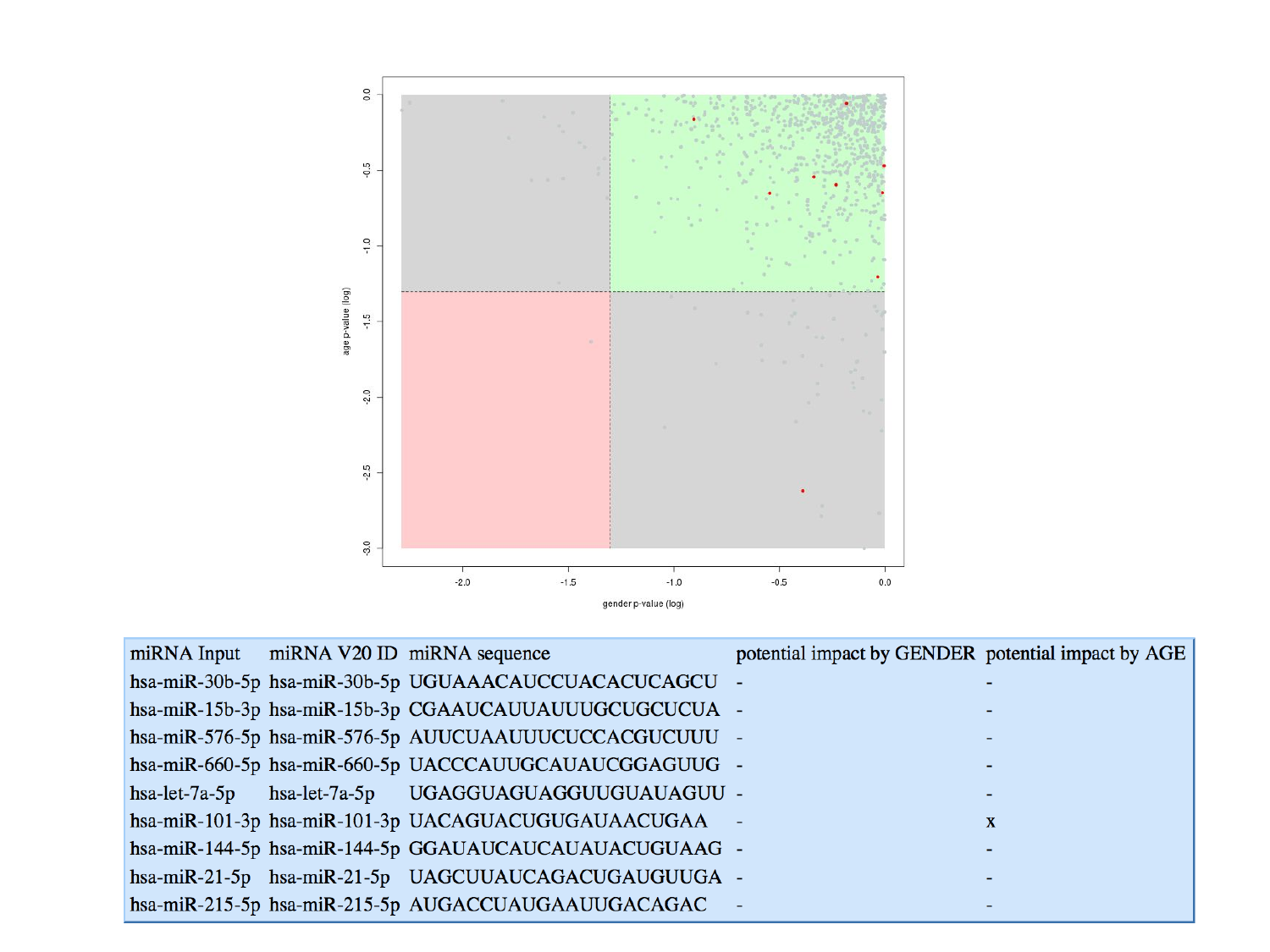

Supplement: Additional file 5: — Results of miRNACon analysis. [file 12974_2015_418_MOESM5_ESM.ppt]
